# Supplementary material for: Real-space observation of ferroelectrically induced magnetic spin crystal in SrRuO3
Source: Nat Commun. 2021 Mar 31;12:2007. doi: 10.1038/s41467-021-22165-5 (PMC8012650; doi:10.1038/s41467-021-22165-5)
Supplement: Supplementary file 1 — Supplementary Information [file 41467_2021_22165_MOESM1_ESM.pdf]

Real-space Observation of Ferroelectrically Induced Spin Crystal in $SrRuO_3$

S. D. Seddon, D. E. Dogaru, S. J. R. Holt, D. Rusu, J. J. P. Peters, A. M. Sanchez, M. Alexe

Department of Physics, University of Warwick, Coventry, CV4 7AL

Supporting Information

Substrate topography

Fig. 1 depicts a typical AFM topography image acquired on a Park XS PSIA instrument in contact mode with a Nanosensors PPP-EFM-50 tip. The line-scan presented in Fig. 1b allows for the determination of step terraces on the substrate of ~200 nm

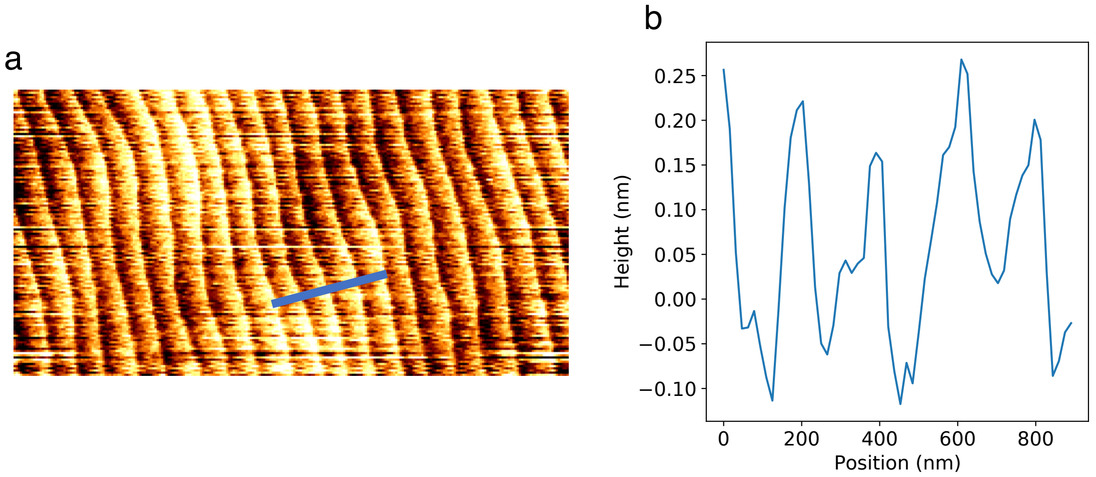

Fig S1: **a** A typical AFM topography, illustrating substrate step terraces of ~200 nm diameter. **b** linescan as marked in **a**

Additional MFM measurements

For completeness, all MFM images acquired are included in figures 2-5. Key features referred to in the main text is the ferromagnetic phase from around 0.3T (Fig. 5) where the periodic domain pattern transitions into a standard domain growth regime, with upward (green) domains saturating into a field polarised state.

During the Fourier analysis, it is noted that the central Fourier peak (not quantified) grew more diffuse over the final switching period, encroaching into the top/bottom edge of the second peak area's integral region. A dynamic pixel window size was adopted to be more focused around peak 2 at these fields, to ensure that the signals integrated were truly from the peak (or indeed the diminishing nature of the peak) and not from this broadening of less periodic noise due to final switching behaviour. All integrals were normalised to the highest signal in Fig. 3b, as well as by pixel area,

allowing all peaks to be compared. Raw data is available on request of the corresponding author.

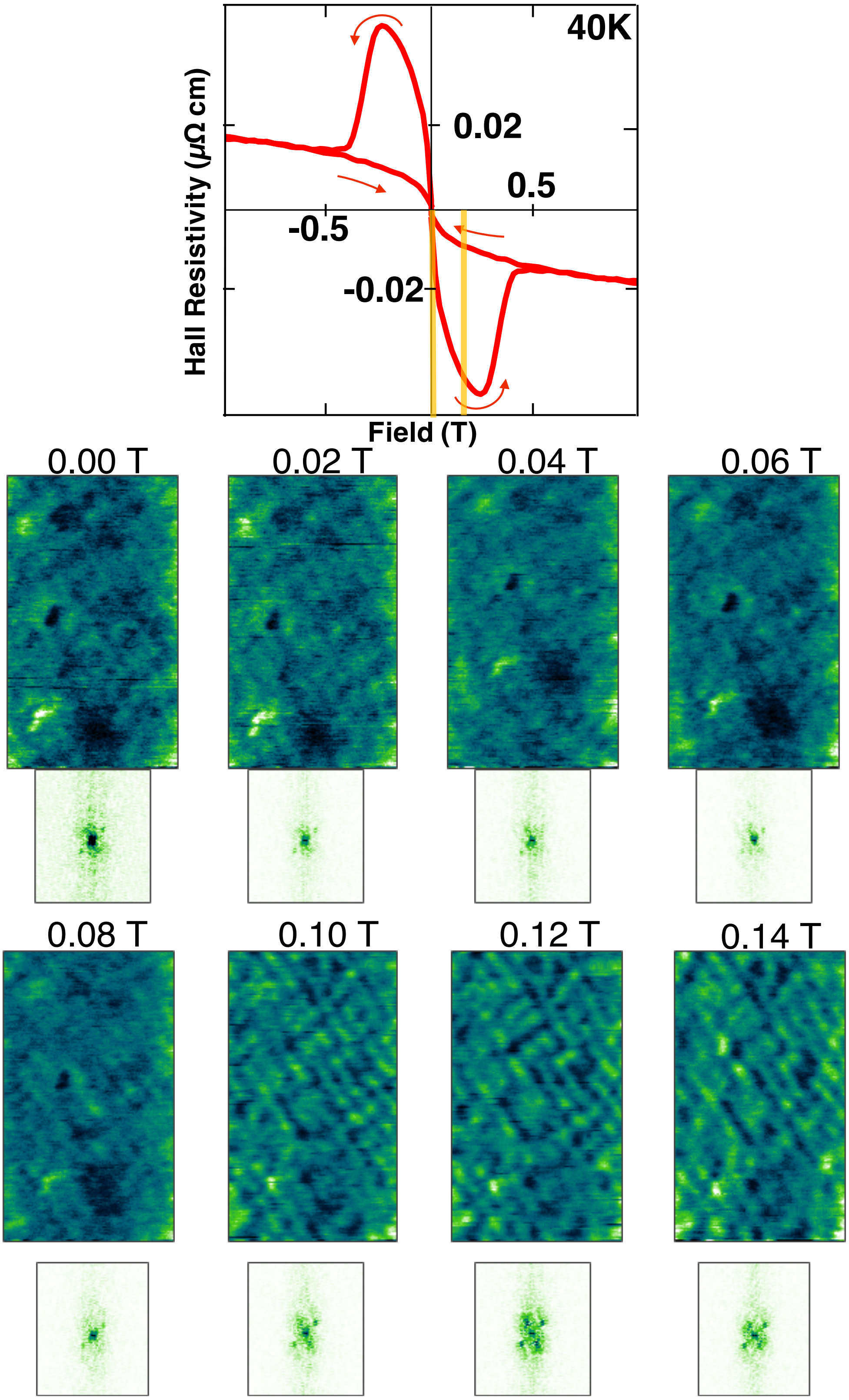

Figure S2: Complete MFM images, fields as marked. Image height is 2.27 μm. Scan direction left to right.

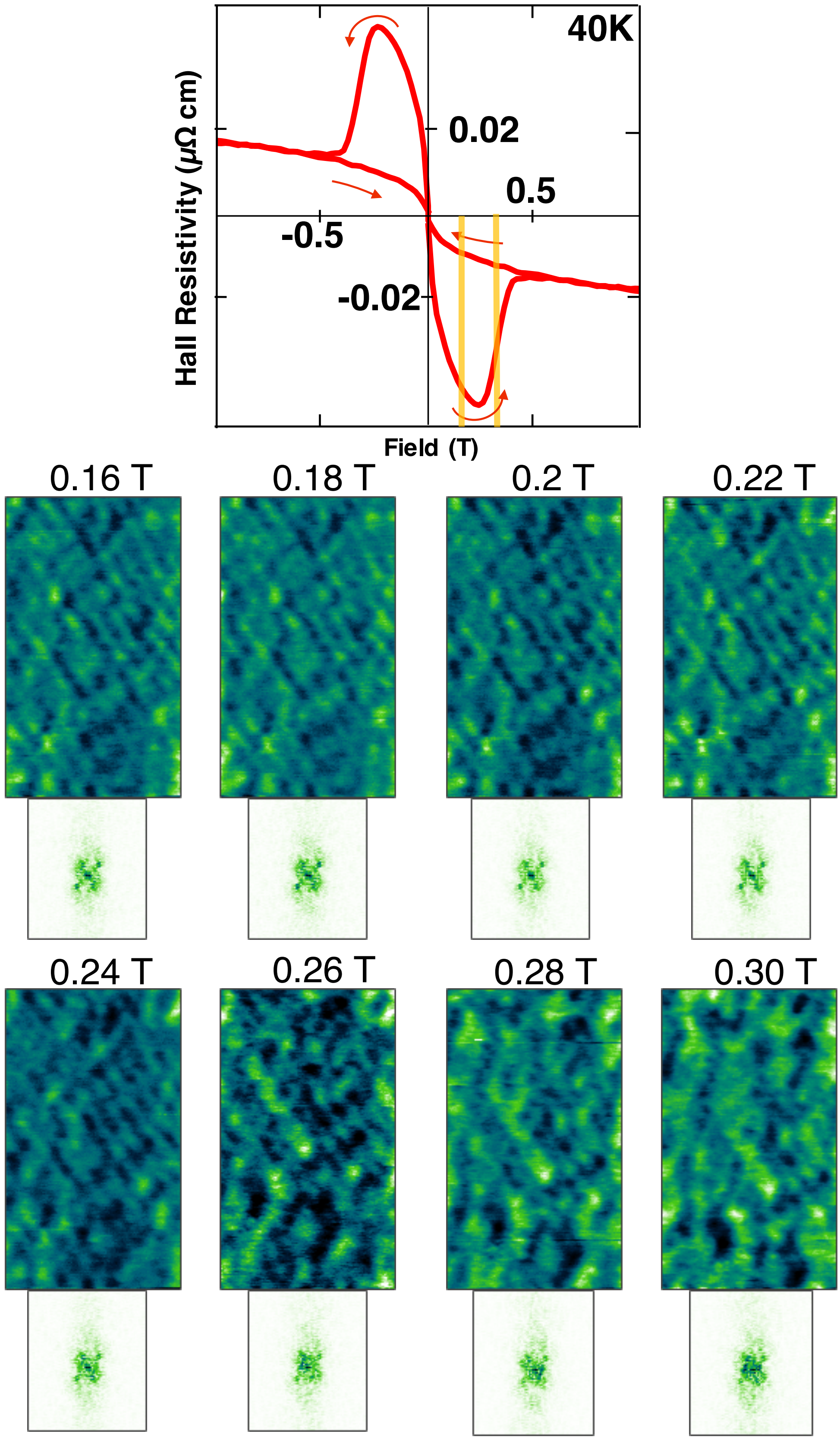

Figure S3: Complete MFM images, fields as marked. Image height is 2.27 μm. Scan direction left to right.

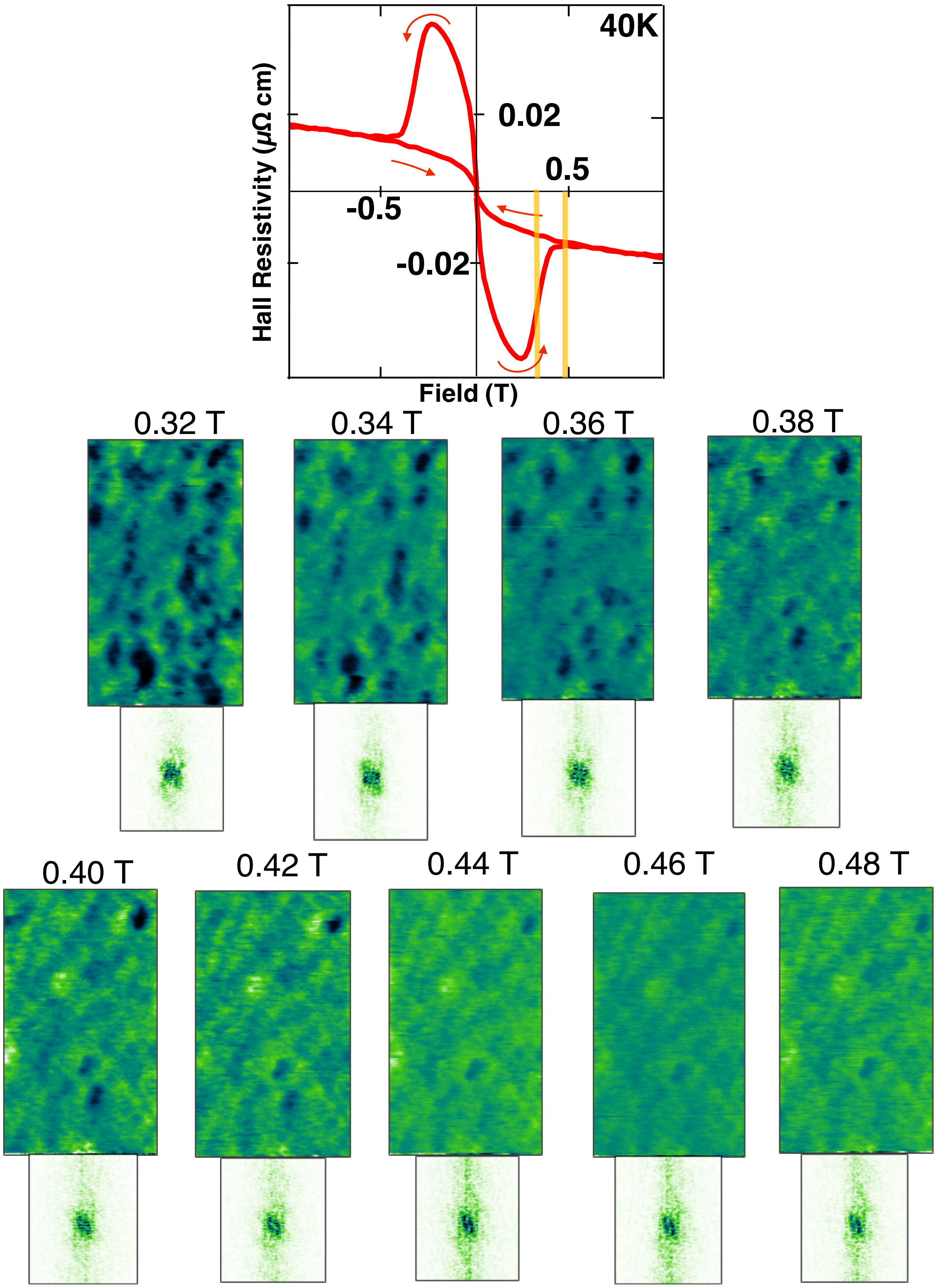

Figure S4: Complete MFM images, fields as marked. Image height is 2.27 μm. Scan direction left to right.

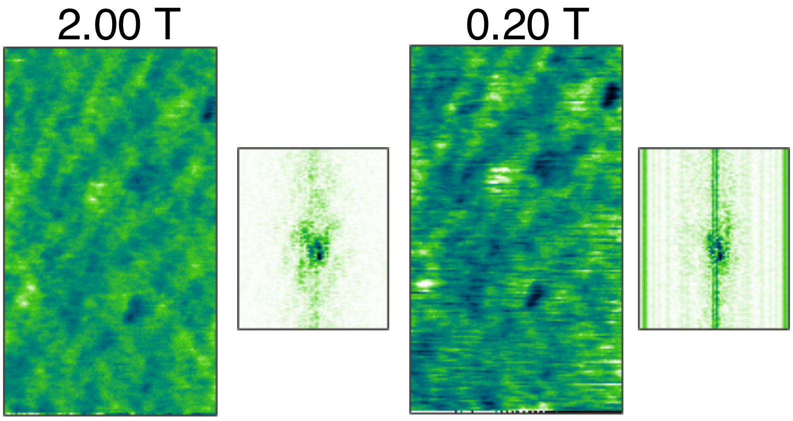

Figure S5: High field image of same area acquired at 2T showing a saturated image, and another image acquired at 0.2T when removing the field, showing no domain formation until complete field removal. Image height is 2.27 μm. Scan direction left to right. Fields as marked
