# Supplementary material for: Real-space observation of ferroelectrically induced magnetic spin crystal in SrRuO3
Source: Nat Commun. 2021 Mar 31;12:2007. doi: 10.1038/s41467-021-22165-5 (PMC8012650; doi:10.1038/s41467-021-22165-5)
Supplement: Supplementary file 3 — Description of Additional Supplementary Files [file 41467_2021_22165_MOESM3_ESM.pdf]

Description of Additional Supplementary Files

Supplementary Movie 1

A sequential series of the MFM measurements as out of plane magneitic field is applied. The relative position in the resistive Hall curve are marked, as are the magnetic phase present. All data acquired at 40K.
